# Supplementary material for: Combination of CENP-B Box Positive and Negative Synthetic Alpha Satellite Repeats Improves De Novo Human Artificial Chromosome Formation
Source: Cells. 2022 Apr 19;11(9):1378. doi: 10.3390/cells11091378 (PMC9105310; doi:10.3390/cells11091378)
Supplement: Supplementary file 1 [file cells-11-01378-s001.zip › cells-1671454-supplementary.pdf]

cells MDPI

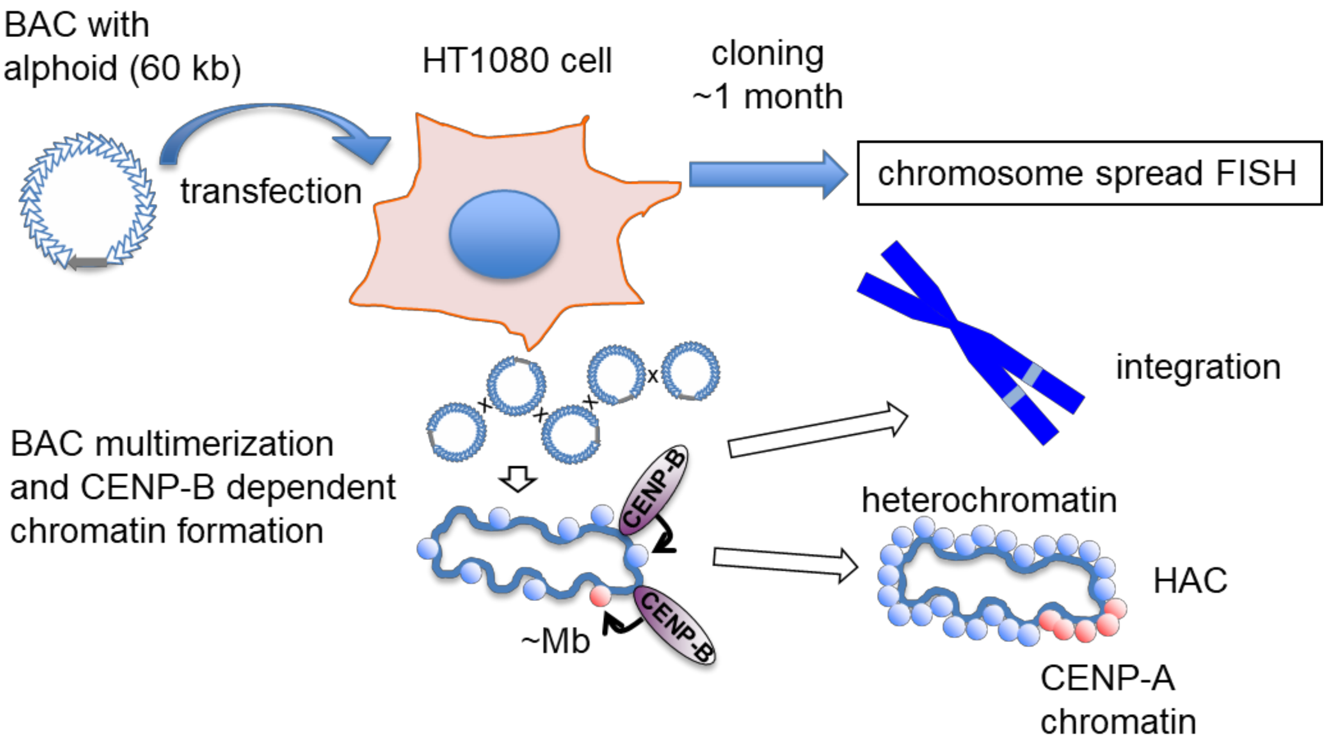

Figure S1. Suggested process for de novo HAC formation.

After introduction into HT1080 cells, the BAC DNA containing large (>30 kb) alphoid repeats multimerizes to megabase order. CENP-B binds to CENP-B box in the alphoid sequence of the multimerized BAC DNA and promotes both CENP-A chromatin and heterochromatin assembly. The balance of these distinct chromatins is important for HAC formation, otherwise the BAC DNA is integrated into the host genome or disappears.

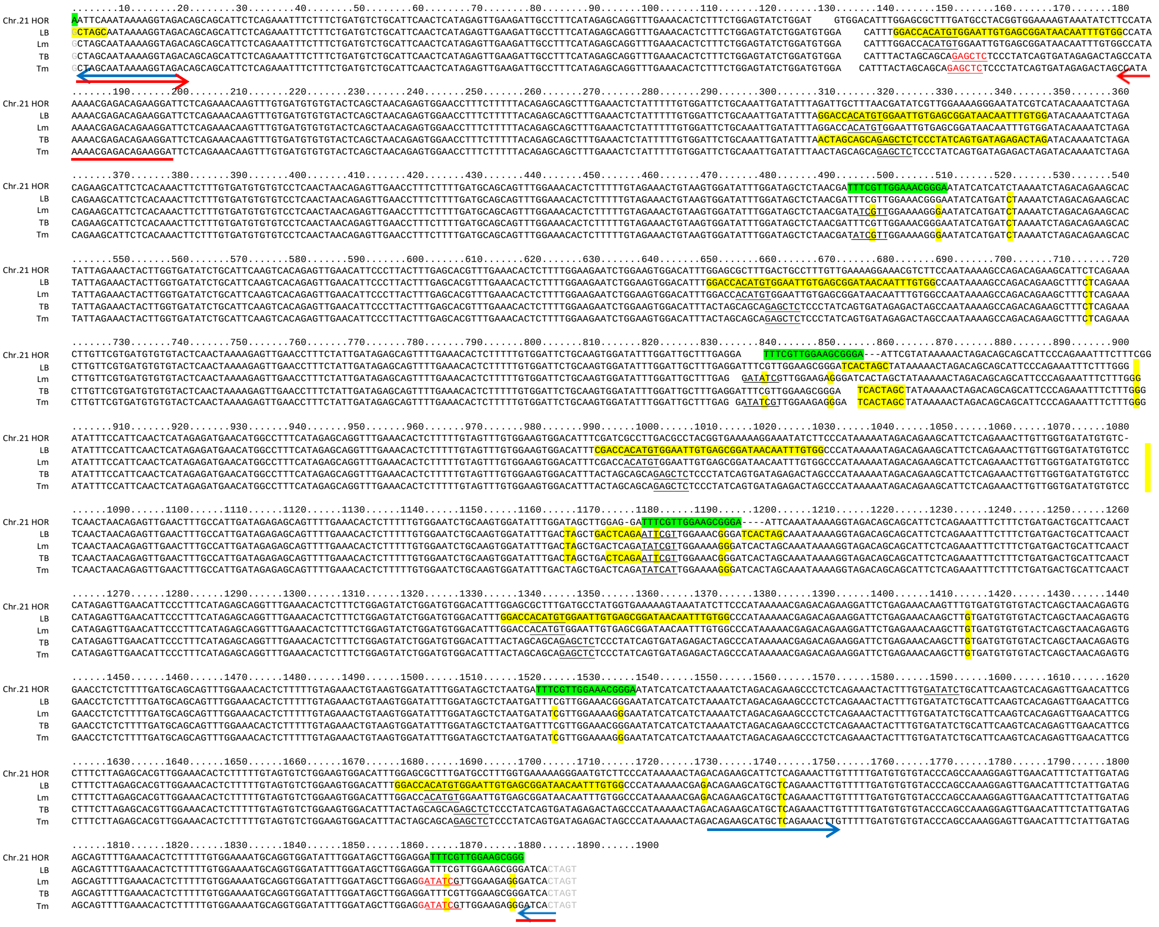

Figure S2. Alignment of the nucleotide sequences of the synthetic alphoids (*Nhe*I to *Spe*I).

Nucleotides to be lost after ligation of the *Nhe*I and *Spe*I sites are presented in gray text. Nucleotides that differ between the five sequences are marked in yellow. CENP-B boxes of 21-I are marked in light green. Positions of the primers for competitive PCR are indicated by arrows (red, lacO vs. tetO; blue, CENP-B box wild-type vs. mutant). Restriction sites introduced in the synthetic alphoids were underlined (red letters, sites used in competitive PCR).

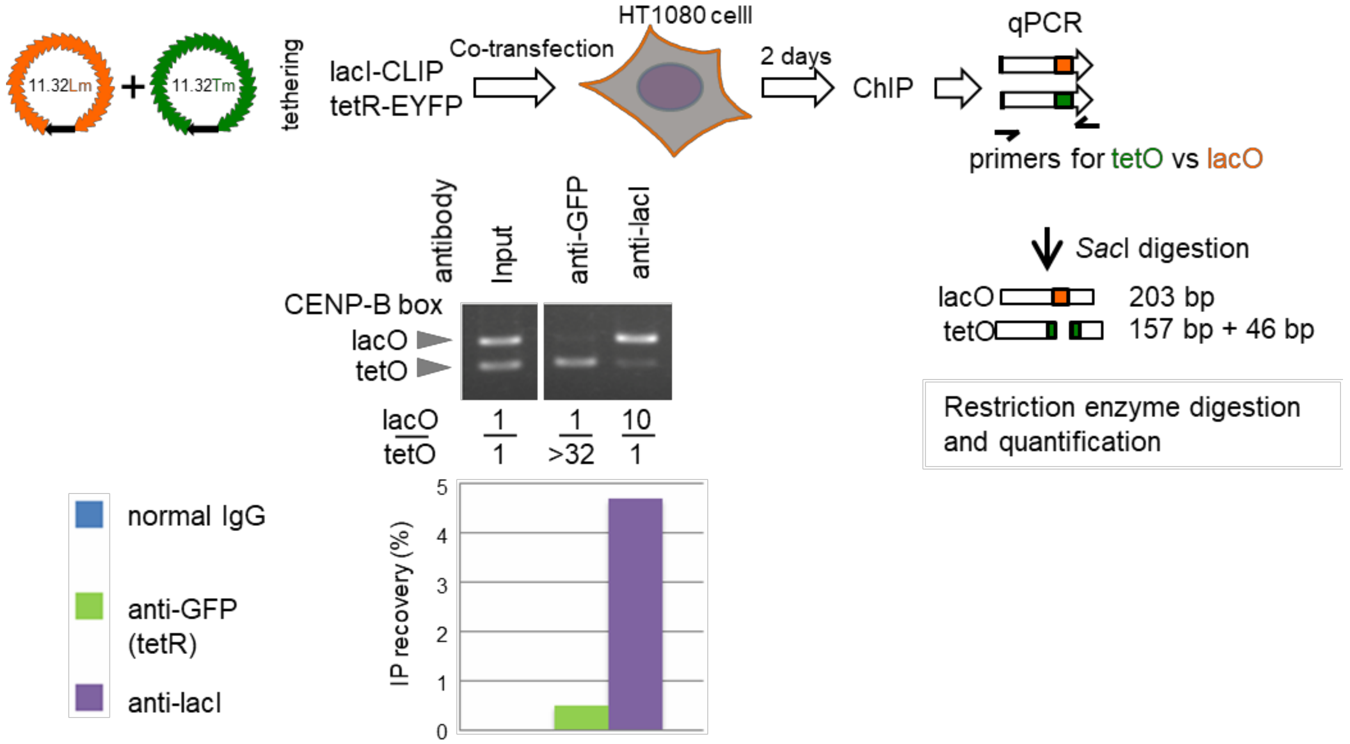

Figure S3. Effectiveness of protein tethering on the synthetic alphoid arrays

The BACs pBAC11.32Lm and pBAC11.32Tm were mixed 1:1 and introduced into HT1080 cells simultaneously with the plasmids expressing lacI-CLIP or tetR-EYFP. ChIP using anti-lacI or anti-GFP (for tetR) was performed after 2 days of transfection. Top, Cartoon of the experiments. Middle, Results of the competitive PCR. Bottom, Results of qPCR. For the competitive PCR, the intensities of the 203-bp bands for the lacO alphoids and 157-bp bands for the tetO alphoids were quantified according to the reference gel (Figure 2c).

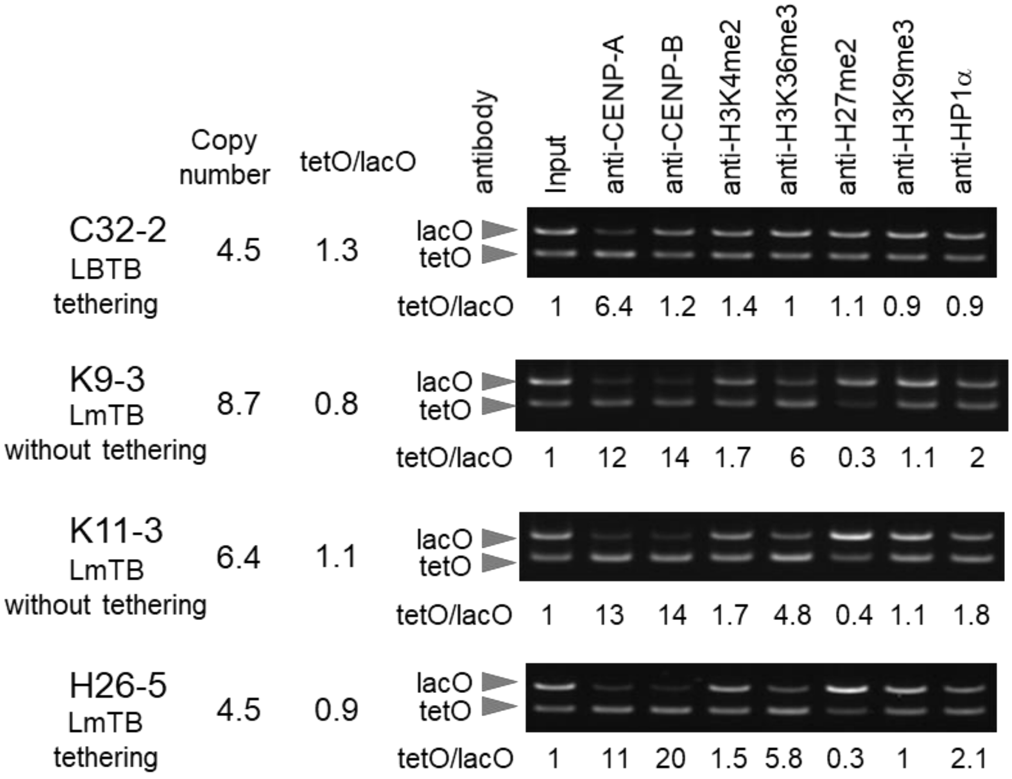

Figure S4. ChIP and competitive PCR analysis of the HAC chromatin structure using synthetic alphoid arrays.

The HAC copy number estimated by comparing the PCR cycles for the synthetic alphoids and 21-I, which is 1.3 Mb in size [51], and the copy number ratios of lacO:tetO for the HACs are shown. The tetO/lacO values described under the electrophoretic images are adjusted for the copy number ratios with lacO:tetO of the HAC as 1.
